# Unique Case Reports of Tooth Sensitivity After Consuming Tetrabenazine

**DOI:** 10.7759/cureus.22318

**Published:** 2022-02-17

**Authors:** Bob Daripa

**Affiliations:** 1 Medicine, Grant Government Medical College and Sir J.J. Group of Hospitals, Mumbai, IND; 2 Internal Medicine: Neurology, Singapore General Hospital, Singapore, SGP

**Keywords:** calcitonin gene-related peptide (cgrp), focal dystonia, adverse drug reaction, tetrabenazine, tooth sensitivity

## Abstract

Tooth sensitivity (TS), a common discomfort manifests as hypersensitivity, sharp and rapid pain, or shock-like sensation in response to tactile, thermal, or chemical stimuli which others perceive as normal, affecting a significant percentage of the population. Tetrabenazine, a vesicular monoamine transport 2 receptor inhibitor acting on presynaptic neuronal endings, is commonly used in acute dystonia treatment. Two cases are reported here, where the patient, after consuming tetrabenazine (TBZ), was noted to have TS after a week of treatment. On stopping TBZ and substituting it with oral anticholinergic, the adverse symptom disappeared and did not recur again. No drugs are yet known to cause TS as an adverse drug reaction, and this is the first report of drug-induced TS cases.

## Introduction

Tooth sensitivity (TS) is a common discomfort affecting 10%-30 population of the 20-50 years age group with a female predominance [1]. It could manifest as hypersensitivity, sharp and rapid pain, or shock-like sensation in response to tactile, thermal, or chemical stimuli, which others perceive as normal, thereby affecting eating and drinking [2,3]. TS has been frequently reported in various studies related to in-office or at-home tooth bleaching procedures using hydrogen peroxide (H2O2), although its presence possesses a hindrance to plaque treatment, thereby compromising oral hygiene [2]. No drugs are yet known to cause TS as an adverse drug reaction (ADR), and this is the first case of drug-induced TS reported here.

Tetrabenazine, a vesicular monoamine transport 2 (VMAT-2) receptor inhibitor acting on presynaptic neuronal endings, is commonly used in acute dystonia treatment [4,5]. Two cases are reported here, where the patients, after consuming tetrabenazine (TBZ), were noted to have TS after a week of treatment. On stopping TBZ and substituting it with oral anticholinergic trihexyphenidyl [5], the bothersome symptoms of TS disappeared and did not recur again after a month’s follow-up.

## Case presentation

Case 1

A young 24 years male diagnosed with drug-induced acute cranial dystonia was treated with oral TBZ at a dose of 25 milligrams (mg) hs which was gradually escalated to 25 mg bid after five days. The patient reported improvement in his facial and neck dystonia but, at the same time, noticed tooth sensitivity severe enough to cause difficulty and discomfort in consuming any food items, be it solid/liquid or hot/cold. Suspecting TBZ as the culprit medication, it was stopped immediately and replaced by oral trihexyphenidyl at a dose of 1 mg at night, gradually escalated to 1 mg bid dose. His TS symptoms disappeared and did not come back.

Case 2

A middle-aged male of 45 years was on anti-depressive and anti-psychotic medications for the past few months. The patient was on oral selective serotonin reuptake inhibitor (SSRI), oral benzodiazepines, and second-generation anti-psychotics serotonin dopamine receptor antagonist, olanzapine. The patient reported acute unilateral focal dystonia of the leg for the last few days with no similar presentation before. Restless leg syndrome was ruled out, and his blood workup of electrolytes, calcium, and creatine phosphokinase (CPK) levels was normal. Suspecting SSRI as the culprit drug, the patient was advised to taper it down for the next few days. At the same time, he was building up tetrabenazine at a dose of 25 mg hs, which was later escalated to 25 mg bid after a week. His dystonic activity lessened, and fortunately, the psychiatric symptoms did not worsen, so finally, it was decided to halt his SSRI gradually. About two weeks, he noted the sensitivity of his tooth, which was gradually increasing to a point where it was difficult for him to chew any solid food items. Tetrabenazine was stopped and changed to oral trihexyphenidyl. Tooth sensitivity in the form of sharp pain with a citrus tinge resolved in a few days and did not recur after one month's follow-up.

## Discussion

Common etiology of tooth sensitivity is enamel loss, use of hard toothbrush and aggressive tooth brushing technique, abrasive ingredients in toothpaste measured in terms of relative dentin abrasivity (RDA), plaque, and periodontal disease, gingival recession, hypo mineralised tooth, or infection of dentin tubules [1,3]. Even fluorosis increases the tooth porosity causing hypo-mineralization of enamel, thereby widening the dentin tubules resulting in a higher prevalence of TS [1,3]. Smoking has conflicting results related to dentin hypersensitivity [3] though the patients in both cases claim that they do not smoke.

Antibiotics like tetracycline, doxycycline, iron and fluoride supplements, an inhaled corticosteroid, and oral mouthwash solutions are well known to cause tooth discoloration but not tooth sensitivity [6]. Certain foods can augment prior tooth sensitivity as sodas, juices, ice cream, citrus fruits, pickled products, or any hot or cold beverages, and therefore should be avoided in TS.

Broadly acknowledged pathomechanism of TS is Brannstrom and Astrom’s ‘hydrodynamic theory’ where external stimulus causes rapid fluid movements in dentinal tubules activating sensory nerves in inner dentin and pulp releasing inflammatory mediators that can depolarize the chemosensitive ion channels TRPA1 causing discomfort, pain, and TS [1,7,8]. The other mechanisms are the direct innervation theory and the odontoblast receptor theory, which are less recognized because of the lack of evidence to support them [9]. As seen in bleaching procedures, where peroxides and their product might inflict an inflammatory process in the pulp region, causing oxidative stress and raising pain mediators, namely prostaglandins, bradykinin, and substance P [2]. This could favour odontogenic pain and tooth sensitivity.

VMAT-2 inhibitor tetrabenazine inhibits mono-amine uptake particularly, dopamine into, presynaptic vesicles. This augments monoamine degradation in neurons into 3,4 di-hydroxy-phenyl-acetaldehyde (DOPAL); 3,4 di-hydroxy-phenyl-acetic acid (DOPAC), and dopamine quinone, further forming hydrogen peroxide (H2O2) through oxidative deamination by monoamine oxidase, hydroxide ion (OH-), and superoxide radical anions (O2-) as stressors [10].

There could be a possibility that these oxidative stressors interfere with the neuronal network in the pulp of the tooth, causing TS [10]. The role of substance P and calcitonin gene-related peptide (CGRP) in TS is not known yet, but their high expression in pulpitis patients has been seen [11]. The role of salivary Ph on the concerned medication metabolism and its relation to dental enamel Ph is also not known. Naranjo ADR probability scale scored 5, which is a probable ADR, and it infers a temporal sequence after a drug intake.

## Conclusions

There is a probability of tetrabenazine causing teeth sensitivity as an adverse drug reaction which is supported by a resolution of symptoms once the offending drug was discontinued. This is a unique presentation which is not been reported before. Since the problem was resolved after stopping tetrabenazine, the patients were not interested in visiting a dentist. Similar cases from neurology or dental clinic in the future would strengthen the above findings. It would also allow exploring the pathophysiology and getting definite answers to the doubtful, possible, or probable theories of TS.
